# Measurement invariance of the English reading motivational structure of bilingual and multilingual university students

**DOI:** 10.1016/j.heliyon.2023.e22884

**Published:** 2023-11-29

**Authors:** Helta Anggia, Andrea Magyar, Anita Habók

**Affiliations:** a Doctoral School of Education, University of Szeged, Hungary; b Center for Research on Learning and Instruction, MTA-SZTE Digital Learning Technologies Research Group, University of Szeged, Hungary; c Institute of Education, University of Szeged, MTA-SZTE Digital Learning Technologies Research Group, Hungary

**Keywords:** Self-related beliefs, Reading motivation, English reading behavior, English reading comprehension, Bilingual, Multilingual

## Abstract

Self-related beliefs can influence language learning motivation. In turn, reading motivation can facilitate self-related beliefs in influencing English reading comprehension (ERC). Additionally, past language learning experience affects future language learning. Thus, this study examined the motivational structure in English reading of 1170 first-year students of 13 universities from nine Indonesian provinces (M_age_ = 19.3, SD = 0.85). Data were collected at one measurement point using the Motivation for Reading Questionnaire to measure reading self-efficacy, extrinsic motivation, and intrinsic motivation (IM); a four-item self-concept questionnaire; the English Reading Behavior Questionnaire, and an ERC. The study developed and evaluated two hypothesized models on the moderating role of past second language (L2) learning experience. The results of structural equation modeling corroborated the moderating influence of being bilingual and multilingual learners on the motivational variables, although not each of the expected paths fit the model. Among the variables, intrinsic motivation exerted the highest effect on the ERC of Indonesian university students. A difference analysis using one-way analysis of variance (ANOVA) on the motivational variables between the two groups demonstrated that multilingual groups outperformed the bilingual group in nearly all motivational variables. The results implied that categorizing students according to their previous learning experience with a foreign language can help teachers to prioritize their teaching to improve the reading comprehension achievement of students.

## 1 Introduction

Since 1990, English has been taught in Grade 4 in primary school in Indonesia. In general, students learn English 70 min in primary and 160 min per week in junior secondary schools [1]. Apart from English, Arabic language also gained prominence among Indonesian students, especially those studying in Islamic-based schools, such as Islamic boarding and public schools (madrasah)*.* The students are motivated to learn the Arabic language to study the religion of Islam [1]. In addition, the mini-survey conducted by the study suggests that, apart from Arabic, the Korean language is increasingly becoming popular among university students in the past few years. The study regards students with only English learning experience as bilingual students and those with learning experience of English and the Arabic or Korean language or both as multilingual students. Furthermore, this study refers to English learning experience as the exposure of students to English learning in their previous education (primary to senior secondary schools) prior to enrolling in university. Although not actively using English in daily communication, these students claim to be proficient in English and able to communicate in spoken and written English. We consider a few of these students as bilingual, because they are proficient in Indonesian and English as native and FLs, respectively. Conversely, a few of the students who claim to be proficient in English were also proficient in the Arabic or Korean language or both. We consider these students as multilingual, because they are proficient in the Indonesian language as their native language and master more than one FL, including English. Referring to the notion that positive language learning experience exerts a positive affective effect on learning the language in the future [2,3], the role of motivation in learning English [4], and the importance of English reading for the academic success of university students [5], we concur that investigating the motivational structure in English reading for students is important.

According to self-determination theory (SDT), the concept of motivation can be understood through, first, the fulfillment of the three human basic psychological needs (BPNS), namely, autonomy, competence, and relatedness [6]. The fulfillment of the BPNS can lead to intrinsic motivation (IM). Second, Deci and Ryan [7] stated that the BPNS are based on needs theory, which posits that needs define the conditions necessary for psychological growth, integrity, and well-being. In other words, the fulfillment of BPNS is important for self-concept, which integrates extrinsically into intrinsically motivated behavior and improves psychological well-being. Third, the different types of motivation, such as amotivation, extrinsic motivation, and intrinsic motivation, exhibit their respective regulatory processes that regulate behaviors and reactions to events such as external, introjected, identified, integrated, and intrinsic regulation. In this regard and on the basis of SDT, the current study understands motivation as a process of fulfilling the BPNS (i.e., autonomy, competence, and relatedness). Thus, investigating the variables that predict the learning motivation of students is crucial and worthy.

Regarding language learning motivation, Grabe [8] states that studies on language learning motivation frequently involve a set of beliefs (self-concept and self-efficacy), values, expectation, and behavior. Although self-concept refers to the belief and evaluation of one's ability, self-efficacy pertains to one's belief in his or her ability to learn and solve a certain task. Prior research demonstrates that self-related beliefs, such as self-concept and SE, predict motivation in learning [9]. In a similar manner, they are the best non-cognitive predictors of academic achievement [[10], [11], [12]]. Self-concept is as important as SE in terms of their relationship with motivation. The reason is that self-concept acts as a source of motivation in relation to the internal view of oneself, whereas self-efficacy triggers one's motivation from his/her belief in his/her capability to accomplish a particular task or challenge [13,14]. Therefore, both variables are important and warrant investigation, especially in the field of foreign language (FL) reading motivation. In theory, FL reading relatively to second language (SL) reading is different in terms of language processing of the readers [15,16]. In other words, the differences of SL and FL students in linguistics knowledge and background knowledge affect their language processing in reading, FL readers tend to have slower language processing and comprehension.

In the case of FL reading, reading motivation plays a key role in facilitating self-concept and self-efficacy with English reading comprehension (ERC) [10] and exerts a positive influence on reading behavior [[17], [18], [19]]. Reflecting on SDT, the study categorizes reading motivation into intrinsic motivation and extrinsic motivation in reading [20]. Although evidence on the facilitating role of extrinsic motivation is scarce, several studies suggested that intrinsic motivation is a strong mediator of self-related beliefs and ERC [[21], [22], [23]]. Similarly, reading motivation has an indirect effect on ERC through reading behavior [24].

Reading amount denotes the reading rate (number of words, paragraphs, or texts) of students as a result of free-reading activities outside the classroom [21]. Day and Bamford [25] propose that extensive reading is voluntary reading conducted outside the context of the classroom for the purpose of increasing the reading amount and reading achievement of students. Additionally, reading amount is one of the aspects of reading behavior. In the current study, extensive reading is crucial, because the reading amount of students investigated here was extracted from self-reported reading activities outside the classroom or extensive reading. The volume of reading amount is expected to positively correlate with improved FL reading comprehension achievement. Therefore, reading amount can be used to investigate FL reading.

Another important variable for investigating FL reading is the effect of a positive language learning experience, which exerts a positive affective effect on learning the language in the future, as explained by motivational self system theory [2,3]. This theory argues that motivation in language learning involves one's effort to reduce the gap between the actual and ideal selves through social experiences in learning. Ewa [26] finds that past language learning experiences exerted an affirmative cognitive influence on learning a new language. However, the question is whether or not past FL learning experiences can also exert a positive influence on the motivation to learn the same or new FLs in the future. We approach this question using the motivational self system theory of Dörnyei [2], which suggests that language learning experience plays a social function for learners in being motivated to learn new foreign language. In this research, we regard the past FL learning experience of students, specifically in the context of Indonesian university students, as exerting a motivational impact when learning the same or a new FL. In terms of its relationship with reading motivation, the past FL learning experience of Indonesian university students is regarded as a moderating variable that may influence their motivational structure difference. Being bilingual or multilingual individual indicates someone's language learning experience. Bilingualism itself is categorized into simalteneous bilingualism (someone could learn two languages at the same time), consecutive bilingualism (someone by any chance learn a language after mastering the other), and receptive bilingualism (someone coul understand a language, but prefer to communicate the most using another language) [27]. On the other hand, multilingual individuals are those who master more than two languages including their native language [28]. As for the dichotomy between bilingual and multilingual individuals, the studies reporting the language processing difference between bilingual and multilingual individuals have been very rare. Instead, there is a cognitive overlap between the two groups, so it is difficult to differentiate between individuals of the two groups in terms of language processing capability [27]. However, Krulatz and Duggan [29] argued that multilingual individuals are stronger than bilingual and monolingual learners in language learning approach.

The majority of previous studies mainly address the effects of self-related beliefs and motivation on reading comprehension [9,10,30], but they mainly overlook the moderating role of past FL learning experiences on ERC and reading motivation [31]. The objective of the study is to determine the structural relationships between self-related beliefs and reading motivation on the reading amount and ERC of university students. The study selects university students, because they have acquired past FL learning experience since primary school and require additional English reading to study their majors in university. Specifically, we examine the facilitating roles of reading motivation, reading amount, and past FL learning experience.

## 2 Theoretical framework

### 2.1 Role of self-related beliefs in English reading

Grabe [8] stated that language learning motivation involves a set of belief (self-concept and self-efficacy), values, expectation, and behavior. Self-concept refers to the belief and evaluation of one's ability [23] and is categorized under academic and non-academic. Academic self-concept is subcategorized to verbal, math, and science self-concept. In native or FL reading, self-concept is included in verbal self-concept [21]. Self-concept is a part of the belief of students that is frequently involved in the discussion on reading motivation.

Self-efficacy is one's belief in one's ability to learn and solve the challenges of a planned task [32]. Reading self-efficacy (SE) is a part of reading motivation that is related to one's ability to address a particular challenge in reading [33]. Schiefele et al. [17] emphasize that readers with high levels of self-efficacy are those who actively face any challenge in reading exercises and plan and attain more satisfactory results relative to those with low levels of self-efficacy. Similar to self-concept, self-efficacy is also always involved in reading motivation studies [14,[33], [34], [35]].

Prior research demonstrated that self-concept has an association with reading motivation [10,21,36]. At the same time, a number of research proposed that self-concept is a strong predictor of reading achievement [22,23,37,38]. Regarding self-efficacy, Yang et al. [14] mentioned that self-efficacy positively influences reading motivation. On the contrary, Caroll and Fox [30] found that self-efficacy only positively influences word reading and ERC but not motivation. Based on previous research, the current study assumed that self-related beliefs are strongly related to reading motivation and ERC [10,14,36]. Moreover, we highlighted that past language learning experiences shape the self-concept and self-beliefs of students.

### 2.2 Role of motivation in English reading

Motivation exhibits a hierarchical classification from being amotivated to extrinsically motivated and finally to intrinsically motivated with varying levels of self-regulation for each stage of motivation [6].

English reading motivation in the context of learners of English as a foreign language (EFL) has mainly been categorized into intrinsic and extrinsic motivation due to the classroom environment, which influences the motivation to read [8]. Previous research has demonstrated the use of both types of motivation to explain motivation among students. For example, Sani et al. [39] has investigated 319 students of English as a second language (ESL) in a Malaysian university using an adapted version of the Motivation for Reading Questionnaire (MRQ). The authors have found that the students were more extrinsically motivated to read to accomplish assignments and to attain the desired reading score. Conversely, several studies have reported that the positive attitude of students toward FL reading was strongly correlated with intrinsic reading motivation and associated the intrinsic reading motivation itself with positive reading achievement [40] using the analytical framework of the Program for International Student Assessment 2018 and the Foreign Language Reading Attitudes and Motivation Scale. In addition, previously recognized questionnaires on the motivation for reading have been constructed using the dimensions of the MRQ. The first is the Motivation for Online Reading Questionnaire (MORQ), which omits several aspects of the MRQ deemed irrelevant to online reading [41]. The MORQ comprises five items organized into four dimensions, namely, curiosity, value, self-efficacy, and self-improvement beliefs. The second is the Adult Reading Motivation Measurement, which is similar to the MRQ in terms of multidimensionality [42]. The questionnaire examines various characteristics of reading motivation, particularly in adolescents. The current study mainly focuses on extrinsic motivation and intrinsic motivation in reading, which were measured using the MRQ.

Another moderating variable may influence the motivational structure of students in English reading and is derived from the L2 motivational self system theory (LMSS) [2]. LMSS is a theory of learning motivation, especially in relation to L2, which postulates learning motivation as a social product [43]. The initial stage of LMSS is L2 learning experience. Specifically, a positive L2 learning experience exerts a positive affective effect on learning the language in the future [2,3]. The present study regards the past L2 learning experience of students to exert a motivational impact when learning a new language. We used LMSS theory to explain the influence of the past L2 learning experience of students on the motivational structure in English reading. Being bilingual or multilingual indicates a person's experience with language acquisition.

Bilingualism is subdivided into simultaneous bilingualism (someone could learn two languages simultaneously), consecutive bilingualism (someone could learn a language after mastering the other), and receptive bilingualism (someone could understand a language, but would prefer to communicate in another) [27]. Multilingual individuals, on the other hand, are those who master more than two languages, including their native tongue [28]. As for the dichotomy between bilingual and multilingual individuals, very few studies have reported differences in language processing between bilingual and multilingual individuals. Instead, there is a cognitive convergence between the two groups, making it challenging to distinguish between individuals of the two groups in terms of language processing ability [27]. However, Krulatz and Duggan [29] argued that multilingual individuals are stronger than bilingual and monolingual learners in language learning approach and how they use the new language they learn.

### 2.3 Reading behavior

Reading behavior is related to reading, which mainly refers to reading amount, text difficulty, book length, and the quality of the reading [21]. Reading behavior can also be an indication of active involvement in reading activities, especially voluntary reading. In the case of ERB, the more internalized the reading motivation is, the more likely the reading activity will bring a sense of enjoyment, which, thus, promotes future reading engagement or positive reading behavior. The concept of future reading engagement is important to reading because reading is less attractive as playing a challenging video game in the majority of cases. If university students want to succeed in their academic achievement, they need sufficient English reading capability to support them in achieving the desired goals [5]. Using their means to inculcate reading habits in themselves, reading behavior is viewed as an authentic proof of reading motivation.

### 2.4 Model development

**Fig. 1:**
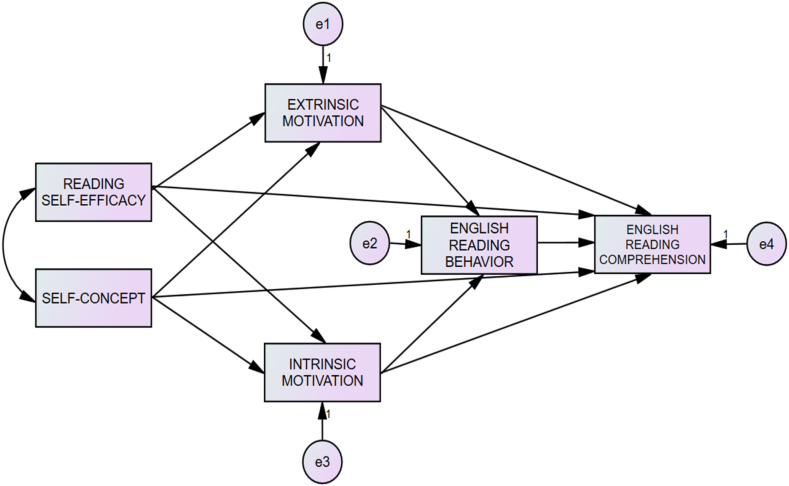
Hypothesized path model of the reading motivation structure.

Based on a review of previous research [2,3,10,14,[21], [22], [23],^26^,^36^,^37^], the current study formulated a hypothesized path model (Fig. 1), which focuses on constructs drawn from previous studies and considers past FL learning experience. As demonstrated by the structural model (Fig. 1), we hypothesize six constructs, namely, two exogenous constructs (self-efficacy and self-concept, which are indicated by the result of the self-report questionnaire) and four endogenous constructs (i.e., extrinsic motivation and intrinsic motivation, which are indicated by the results of the self-report questionnaire), and ERB (which is indicated by the report of the students on reading amount, length, and frequency). The last endogenous construct is ERC, which is indicated by the result of an ERC test. Moreover, we hypothesize 11 structural relationships, namely, the direct effects of self-efficacy and self-concept on extrinsic motivation intrinsic motivation, and ERC constructs, of extrinsic motivation and intrinsic motivation on ERB and ERC, and of ERB on ERC.

**Fig. 2:**
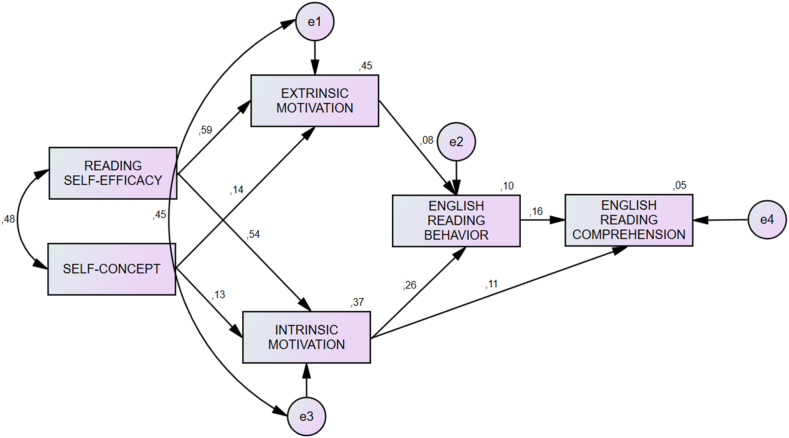
Motivational structure of bilingual students.

**Fig. 3:**
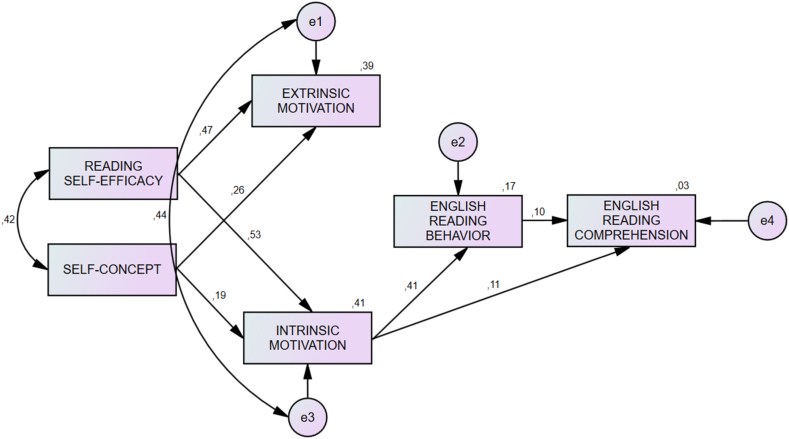
Motivational Structure of Multilingual students.

### 2.5 Objectives of the present study

The present study is based on the assumption that self-related beliefs can influence language learning motivation and that motivation plays an important role in English learning [8,44]. In addition, the study highlights that the importance of English reading is inevitable to the academic success of university students [5,21]. Moreover, positive language learning experiences have a positive influence on future language acquisition [2,3]. Similarly, Ewa [26] found that past L2 learning experience exerts an affirmative cognitive influence on learning L3. The question is whether or not past FL learning experience can also exert a positive influence on learning of the same or other FLs in the future in which L2 learning experience influences the same L2 or L3 learning motivation. We approach this question using the L2 motivational self system theory of Dörnyei [2] with L2 learning experience. The theory mentioned L2 learning experience as a social function that enable learners to be motivated to learn L2. The present study hypothesizes that a student's previous L2 learning experience has a motivational effect on the acquisition of a second language. We used LMSS theory to explain the effect of prior L2 learning experience on the motivational structure of English reading. Language acquisition experience is indicated by a person's bilingual or multilingual status. Therefore, grouping the students to bilingual and multilingual group of students is important to determine the differences in the reading motivational structures of both groups.

We intend to elucidate the interrelationship among these variables and identify the possible pedagogical influence of the model on the reading comprehension of bilingual and multilingual students. On the basis of the abovementioned theories on self-related beliefs, motivation, reading behavior, and past L2 learning experience, this study intends to answer the following research questions.

RQ1 What is the extent to which self-related beliefs and the types of motivation of bilingual and multilingual students directly and indirectly correlate with text comprehension?

RQ2 What is the extent to which the relationships described in the structural models (RQ1) vary across bilingual and multilingual students?

## 3 Method

### 3.1 Participants

The study recruited 1170 first-year students from 13 universities across nine Indonesian provinces. The mean age of the students was (*M* = 19.3, *SD* = 0.85). The participants were classified into two groups, namely, bilingual and multilingual. A total of 595 students claimed to be bilingual (having experience with L1 and English learning). We considered a few of the students to be bilingual, because they were proficient in Indonesian and English as their native and foreign languages, respectively. The other 575 students claimed to be multilingual (with experience with L1, Arabic or Korean language, and English learning). We considered these students as multilingual students, because they were proficient in the Indonesian language as their native language and mastered more than one FL, including English. The students were enrolled in social and science majors and previously learned English for a span of six to ten years.

### 3.2 Instruments

This study employed several instruments, such as a few items for academic self-concept, the MRQ to examine reading motivation (i.e., self-efficacy, extrinsic motivation and intrinsic motivation), the questionnaire on ERB, and an ERC test.

First, the items for academic self-concept were derived from García-Grau [45]. The four items highlighted the perceptions of students and their assumption of the perception of teachers about their academic self-concept (i.e., I am a good student; My teachers think I am a good student). Each item was rated using a four-point response scale, namely, *very different from me* = 1, *a little different from me* = 2, *a little like me* = 3, and *a lot like me* = 4. The reliability of the items was very high (Cronbach's α = 0.97). These items of the self-concept fit the construct of self-concept in that it refers to the belief and evaluation of one's ability [23]. In this case, the students view and value themselves as learners.

Second, the study adopted the MRQ by Wigfield and Guthrie [46] for measuring self-efficacy, intrinsic motivation, and extrinsic motivation The MRQ is an established instrument for reading motivation, which has been validated in various contexts. It presents 11 constructs under three major categories, namely, competence beliefs (self-efficacy), intrinsic and extrinsic reading motivation, and social reason for reading. In the Asian context, Wang and Jin [47] validated the MRQ on 522 students in the seventh to ninth grades and found that intrinsic motivation and extrinsic motivation were the two constructs with high factor loadings. Self-efficacy also loaded significantly. Wang et al. [48] investigated adolescent students in China using the MRQ instrument and emphasized the importance of intrinsic motivation for reading. The samples of the current study are university students; thus, we only used the most relevant 19 items of the MRQ. We selected questions that matched the relevance of the sample as older learners. Out of the 19 items selected, we chose five items for self-efficacy (e.g., I am a good reader), ten items for intrinsic motivation (e.g., I read about my hobbies to learn more about them), and four items for extrinsic motivation (e.g., I read to improve my grades). The items were rated using a four-point scale, namely, *very different from me* = 1, *a little different from me* = 2, *a little like me* = 3, and *a lot like me* = 4. The reliability of the items was very high (Cronbach's α = 0.915).

Third, the items for ERB were adopted from Wang et al. [24]. The three items focused on the reading amount (number of books read for interest during the previous month; 1 = *0 books*; 2 = *1–2 books*; 3 = *3–4 books*; 4 = *more than 5 books*), reading length (time usually spent on reading a book without taking a break when reading for interest; 1 = 5 min; 2 = 15 min; 3 = 30 min; 4 = 60 min *or more*), and reading frequency (how often they read for interest; 1 = *almost never*, 2 = *once a month*, 3 = *once a week*, and 4 = *almost every day*). The reliability of the items is (Cronbach's α = 0.98), and the objective of these items matches the theory that the behavior of students toward reading can be indicated by reading quantity, such as reading amount, time invested in reading, and reading frequency [21].

Fourth, the items for ERC were adopted from the Common European Framework of Reference for Languages test of EF English First. We used 20 multiple-choice questions to evaluate five cognitive processes when comprehending an English text, namely, identifying the main ideas (i.e., 5. What is the main idea of the passage?), locating detailed information (i.e., 10. When did tablet technology first appear on television?), inferring (i.e., 12. From the passage we can infer that …), and answering vocabulary questions (i.e., 16. The word “greasy” in the first line of the second paragraph is closest in meaning to …). The reliability of the reading test reached Cronbach's α = 0.70. The readability level of the texts in the reading test was adjusted according to the English proficiency levels of the students (i.e., A1–C1) [49].

### 3.3 Design

The Institutional Review Board of the Doctoral School of Education at the University of Szeged authorized the study. A total of 1170 participants provided informed consent. With the aid of their instructors, the participants filled out the Google Form questionnaires for the variable and completed the ERC test. Furthermore, under the guidance of their instructors, the students spent 60–70 min to complete the questionnaires and the test. The teachers at each university spent a few minutes explaining the specifics of completing the questionnaire and the ERC test.

### 3.4 Data analysis

The first analysis was conducted to determine the reliability of all instruments. Although self-concept displayed a very high reliability (Cronbach's α = 0.97), the MRQ, which consists of self-efficacy, intrinsic motivation, and extrinsic motivation obtained a very high reliability (Cronbach's α = 0.915). Next, the reliability of the ERB instrument reached Cronbach's α = 0.98. Finally, the reliability of the ERC test was Cronbach's α = 0.70. Afterward, the study conducted correlational analysis to determine the correlation among the variables (self-concept, self-efficacy, intrinsic motivation, extrinsic motivation, ERB, and ERC). Correlational analysis was conducted on the two groups of students [50]. The correlation was significant at the 0.01 level (2-tailed). The second analysis was a *t*-test analysis to identify the differences for each motivational variable. The *t*-test is considered significant at p-value <0.05. Both analyses were conducted using SPSS 25 version. The third analysis was employed a multigroup structural equation model (MSEM) for motivational structure using AMOS 23 [51]. This analysis observed the fit indices of both models, such as comparative fit index (CFI >0.9), Tucker Lewis index (TLI >0.9), normed fit index (NFI >0.9), and root mean square error of approximation (RMSEA <0.06) [52]. Furthermore, the study also intended to analyze the decomposition of effect in the structural path. The effect is significant at p-value <0.01. The last aspect observed in the MSEM was testing the invariance of the structural model across bilingual and multilingual groups at p-value <0.01.

## 4 Results

The study conducted correlational analysis to determine the correlation among the variables for both groups. The variables were ERB, intrinsic motivation, extrinsic motivation self-efficacy, self-concept, and ERC. Nearly all variables for the bilingual group were significantly correlated, except for EM with ERC (*r* (595) = 0.052, *p* > 0.001), self-efficacy with ERC (*r* (595) = 0.058, *p* > 0.001), and self-concept with ERC (*r* (595) = 0.047, *p* > 0.001). The same is true for the multilingual group in which all variables were significantly correlated except for extrinsic motivation with ERC (*r* (575) = 0.004, *p* > 0.001), self-efficacy with ERC (*r* (575) = 0.072, *p* > 0.001), and self-concept with ERC (*r* (575) = 0.039, *p* > 0.001). The most notably correlated variables in the bilingual group were extrinsic motivation with intrinsic motivation (*r* (595) = 0.678, *p* < 0.001), self-efficacy with intrinsic motivation (*r* (595) = 0.601, *p* < 0.001), and self-efficacy with extrinsic motivation (*r* (595) = 0.662, *p* < 0.001). At the same time, the most notably correlated variables in the multilingual group were extrinsic motivation with intrinsic motivation (*r* (575) = 0.662, *p* < 0.001), self-efficacy with intrinsic motivation (*r* (575) = 0.613, *p* < 0.001), self-efficacy with extrinsic motivation (*r* (575) = 0.582, *p* < 0.001), and self-concept with extrinsic motivation (*r* (575) = 0.456, *p* < 0.001). The rest of the variables was also significantly correlated, but the correlation was less than 0.400 (Table 1).

**Table 1:** Correlation among variables in the motivation structure of bilingual and multilingual groups.

English Reading Behavior (ERB)	ERB	IM	EM	SE	SC	ERC
	1	0.315^a^	0.258^a^	0.244^a^	0.155^a^	0.191^a^
Intrinsic Motivation (IM)	0.410^a^	1	0.678^a^	0.601^a^	0.391^a^	0.156^a^
Extrinsic Motivation (EM)	0.253^a^	0.662^a^	1	0.662^a^	0.427^a^	0.052
Reading Self-Efficacy (SE)	0.279^a^	0.613^a^	0.582^a^	1	0.477^a^	0.058
Self-Concept (SC)	0.216^a^	0.416^a^	0.456^a^	0.416^a^	1	0.047
English reading comprehension (ERC)	0.145^a^	0.155^a^	0.004	0.072	0.039	1

a Correlation is significant at the 0.01 level (2-tailed). Upper side for bilingual and downside for multilingual correlations.

The study conducted a difference analysis to compare the bilingual and multilingual groups in terms of all variables using an independent sample *t*-test (Table 2). First*,* the study noted a significant difference for self-efficacy (*t* (575) = −7.878, *p* = 0.000) in which the multilingual group (*M* = 6.00, *SD* = 1.128) attained higher levels of self-efficacy than those of the bilingual group (*M* = 6.5.41, *SD* = 1.414). Second*,* a significant difference is noted for English reading behavior (*t* (595) = 59.05, *p = 0.000*). The bilingual group (*M* = 80.74, *SD* = 30.89) attained higher scores on the ERB compared with those of the multilingual group (*M* = 5.80, *SD* = 1.776). Third, the study found a significant difference for intrinsic motivation (*t* (575) = −9.970, *p* = 0.000), where the multilingual group (*M* = 39.96, *SD* = 5.832) attained higher levels of intrinsic motivation than those of the bilingual group (*M* = 36.01, *SD* = 7.629). Fourth*,* a significant difference was observed for extrinsic motivation (*t* (575) = −10.593, *p* = 0.000) in which the multilingual group (*M* = 11.88, *SD* = 2.036) obtained higher levels of extrinsic motivation than did the bilingual group (*M* = 10.31, *SD* = 2.942). Fifth, the results indicated a significant difference for self-concept (*t* (575) = −12.853, *p* = 0.000) with the multilingual group (*M* = 11.96, *SD* = 3.293) attaining higher levels of self-concept than those of the bilingual group (*M* = 9.65, *SD* = 3.632). Sixth, a significant difference existed for ERC (*t* (575) = −81.916, *p* = 0.000), where the multilingual group (*M* = 11.96, *SD* = 3.293) attained higher scores for ERC than did the bilingual group (*M* = 0.59, *SD* = 0.493). The only variable in which the bilingual group achieved higher levels than did the multilingual group was for ERB.

**Table 2:** Differences in motivational variables between the bilingual and multilingual groups.

Variable	Language	*n*	*M*	*SD*	*F(2.1168)*	*p* <
ERB	0	595	80.74	30.899	3371.235	0.001
	1	575	5.80	1.776	98.520	
Intrinsic motivation	0	595	36.01	7.629	110.868	0.001
	1	575	39.96	5.832	61.600	
Extrinsic motivation	0	595	10.31	2.942	163.076	0.001
	1	575	11.88	2.036	6933.374	
Reading self-efficacy	0	595	5.41	1.414	3371.235	0.001
	1	575	6.00	1.128	98.520	
Self-concept	0	595	9.65	3.632	110.868	0.001
	1	575	11.96	2.435	61.600	
English reading comprehension	0	595	0.59	0.493	163.076	0.001
	1	575	11.96	3.293	6933.374	

0 = Bilingual group, 1 = Multilingual group.

Before testing the invariance of the structural model between bilingual and multilingual groups, we tested the goodness-of-fit of both models. The structural path model of the multigroup models and the bilingual and multilingual groups exhibited a good fit (Table 3). The study used the MSEM for motivational structure to determine the effect of each variable on ERC as the dependent variable. This analysis also intended to identify which coefficient correlations were significant between the two moderating groups. Prior to structural testing, we decomposed the effects of the variables on the structural path. Based on the decomposition of effects, intrinsic motivation accounted for individual differences in ERC for the bilingual (standardized path coefficient = 0.232, *p* < 0.01) and multilingual (standardized path coefficient = 0.268, *p* < 0.01) groups. In addition, ERB accounted for individual differences in ERC for both groups (bilingual: standardized path coefficient = 0.164, *p* < 0.01; multilingual groups: standardized path coefficient = 0.094, *p* < 0.01; Table 4).

**Table 3:** Structural equation modeling analysis: Model fit statistics.

Model	Description	*ꭕ*^2^	df	p	CFI	TLI	NFI	RMSEA
Multigroup model	Intervention model of motivation and ERB	4.740	4	0.315	1.000	0.997	0.998	0.013
Model 1 (Bilingual group)	Intervention model of motivation and ERB (Fig. 2)	6.156	5	0.291	0.999	0.997	0.994	0.020
Model 2 (Multilingual group)	Intervention model of motivation and ERB (Fig. 3)	13.759	6	0.032	0.992	0.979	0.986	0.047

**Table 4:** Decomposition of effects for structural paths.

Path	Direct effect	Indirect effect	Total effect	Correlation
	Bilingual group (*N* = 595)			
	Dependent variable: English reading comprehension			
Reading self-efficacy	−0.034	0.074	0.041	0.058
Self-concept	0.006	0.019	0.024	0.047
Extrinsic motivation	−0.099	0.013	−0.085	0.052
Intrinsic motivation	0.190	0.043	0.232	0.156**
English reading behavior	0.164	0.000	0.164	0.191**
	Multilingual group (*N* = 575)			
	Dependent variable: English reading comprehension			
Reading self-efficacy	0.009	0.058	0.067	0.072
Self-concept	0.001	0.005	0.007	0.039
Extrinsic motivation	−0.177	−0.003	−0.180	0.004
Intrinsic motivation	0.228	0.041	0.268	0.155**
English reading behavior	0.094	0.000	0.094	0.145**

Afterward, the study tested the invariance of the structural model between the bilingual and multilingual groups. The relationships among self-efficacy, self-concept, intrinsic motivation, extrinsic motivation, ERB, and ERC were equivalent across both groups except for the coefficient paths between self-efficacy and extrinsic motivation (*p* < 0.01, Z-score = −3.937) and between intrinsic motivation and ERB (*p* < 0.01, Z-score = 3.043; Appendix 1).

## 5 Discussion

This study investigated the reading motivation of EFL university students and its facilitating role in the relationship between self-related beliefs and ERC achievement. Additionally, the study investigated whether or not reading motivation has an indirect effect on ERC through ERB. One of the objectives of the study was to compare the English reading motivational structures of both bilingual and multilingual groups. Consequently, this study addressed the data in relation to the abovementioned research questions.

### 5.1 RQ1

One of the questions that this study was aiming to answer was the extent to which self-related beliefs and the types of motivation of bilingual and multilingual students directly and indirectly correlate with text comprehension. The result, that is, the low levels of direct and indirect association of the self-efficacy and self-concept of both groups to ERC, is in line with that of Caroll and Fox [30]. A possibility exists that an encouraging intervention is required to facilitate self-efficacy to optimize ERC. In other words, repeated practice is needed to form the habit of reading among the students.

Intrinsic motivation, instead of extrinsic motivation positively facilitated the association of self-efficacy and self-concept to ERC for both groups (Figs. 2 and 3). This finding confirms the statement of Ryan and Deci [6], that the more autonomous a learner is, the more internal the motivation for learning becomes. The significant function of the facilitating role of intrinsic motivation is also in line with the proposal of [10], which found that intrinsic motivation facilitated the relationship between self-related beliefs and reading achievement. On the other hand, extrinsic motivation negates the intervention role of motivation in the effect of self-efficacy and self-concept on ERC (Figs. 2 and 3). This finding confirms the result of Habók's [10] in which instrumental motivation does not facilitate personal traits or self-related beliefs with ERC. Instead, it denies the argument of Yang's et al. [14] that extrinsic motivation can facilitate the relationship between self-related beliefs and ERC. However, the analysis proves that extrinsic motivation is existentially important to influence the effects of intrinsic motivation on ERB and ERC. Although the current study noted a difference in the relationship between extrinsic motivation and ERB for both groups (Figs. 2 and 3), we believe that extrinsic motivation co-contributed to the relationship between intrinsic motivation and reading behavior. Therefore, we considered extrinsic motivation as important to both path models. In this regard, future studies should test the reciprocal relationship between self-related beliefs and motivational aspects [9]. As such, enhancing self-efficacy and self-concept through intervention will help facilitate the motivation of students in ERC [10]. This study's findings indicate that future reading instruction must incorporate extrinsic motivation in the form of external rewards for students' reading accomplishments. External rewards for students cannot be ignored under any circumstances.

Based on the ERC scores, the intrinsically motivated students were proven to outperform those who were extrinsically motivated. This fact specified the relationship between motivation and reading comprehension achievement [[53], [54], [55]]. Intrinsic motivation was more dominant than extrinsic motivation in influencing ERC. However, Ryan and Deci [6] stated that a controlling aspect may exist in motivation although it is internal. Therefore, conducting an in-depth investigation of the perceived locus of control of the intrinsic motivation of students as a future step of this study would be illuminated for the future.

The finding of the current study confirmed the facilitating role of reading behavior as manifested by the English reading amount, length, and frequency outside the classroom, although it was non-significantly high (Figs. 2 and 3). This result supports those of Wang et al. [24] in which reading amount, length, and frequency can help motivate readers to enhance their reading comprehension achievement. Moreover, although it does not significantly influence ERC, ERB outside the classroom can be an option for university teachers to enhance the English reading ability of their students. The coefficient paths of ERB to ERC for both groups confirmed the cognitive association between ER and ERC, which is similar to the results of Ng's et al. [56], who found that avid readers in an extensive reading program managed to boost reading scores only by reading whatever they like to read as much as possible. However, the finding of the current study provides evidence that the cognitive effect of ERB on ERC was at a low level and may take time and demand the determination of students and teachers. Future extensive reading intervention for enhancing students' English reading comprehension should be well managed and continuously conducted for a long-term objective.

### 5.2 RQ2

Prior to the study, the researchers anticipated that the multilingual group would outperform the bilingual group [26]. We also assumed that variance in motivational structure existed between the two groups given that the L2 learning experience of multilingual students may have placed them on an affectively higher level than the bilingual group. Prior L2 experience may have influenced the motivation of the students to study L2, particularly their self-concept [9]. As self-concept is one's evaluation of one's academic ability on the basis of past learning experience [21,23], we, therefore, hypothesized that prior L2 learning experience was substantially related to self-concept. The research on language learning motivation frequently includes other variables of motivation such as self-efficacy, intrinsic motivation, extrinsic motivation and ERB [[10], [11], [12], [13], [14]]. To explain the result of the current study regarding the motivational variable invariance of both groups, we used L2 motivational self system theory in relation to the moderating role of past L2 experience.

The moderating role of FL learning experience did not seemingly vary the motivational structure, because both tested models only exhibited variance in the self-efficacy – extrinsic motivation and intrinsic motivation–ERB paths. This result partially denied the assumption of the self-motivational system [2,3] on the effect of past L2 learning experience on new FL learning achievement and motivation. The self-efficacy of the bilingual group tended to display a more association with extrinsic motivation than did the multilingual students. At the same time, the intrinsic motivation of the multilingual students exhibited a more association with ERB than that of the bilingual group (Appendix 1). Past FL experience could be only helpful for the cognitive aspect, instead of the motivational aspect, of FL [26]. However, the difference analysis on the variables of both groups indicated that, in summary, the motivational variables of the multilingual group were better than those of the bilingual group. This finding supported our argument that prior FL learning experiences exert an affective influence when learning another FL in the future [2,3,57]. According to the L2 motivational self system theory, motivation in language acquisition involves the effort to fill the gap between the actual and ideal selves through social experience in learning. The findings of the current study indicated that prior L2 classroom and social learning experiences enhances one's motivation to reach the ideal self in learning, which positively influences one's motivational variables such as self-related beliefs, intrinsic motivation, extrinsic motivation, and ERB. These motivational variables can be the source of someone's learning approach in language learning. The language learning approach is determined by the extent of language learning experience one might have. Our finding confirmed what Krulatz and Duggan [29] argued that multilingual individuals benefit from their language learning experience that affects their language learning approach. Thus, in case of foreign language reading such as English reading, multilingual students have better self-concept and self-efficacy, and more intrinsic motivation to read. Their language learning experience makes them believe that they are gifted in English reading. The past language learning experience also builds their belief in their English reading capability. As a result, they become intrinsically more motivated in reading, thus resulting in a better English reading comprehension achievement relative to bilingual students and monolingual students whose first language is other than English. As a result of this statistical finding, we recommend that students study as many foreign languages as possible. Foreign language study provides students with more practical experience and greater motivation to study another language in the future. In the case of English reading, more experience studying a foreign language can have a positive effect on reading motivation.

## 6 Conclusion and limitation

In accordance with the objectives of the study, we aimed to elucidate the educational views required of teachers and researchers in FL reading motivation. We anticipate that by holding the beliefs suggested by the findings, teachers would be able to commence progressive instructional improvement to enhance the ERC of students. The motivational models for both groups demonstrate only significant variance in the self-efficacy – extrinsic motivation and intrinsic motivation–ERB paths. The multilingual group differs significantly from the bilingual group in nearly every aspect of motivational structure in FL reading. This study contributed to the field of non-cognitive factors in FL reading by illustrating the probable existence of differences in motivation between those with less FL learning experience (only mastering one FL apart from the native language) and with more FL learning experience (mastering more than one FL) in English reading motivation. Clinical intervention in every motivational aspect for both final models is required to facilitate both groups in achieving the desired outcome of the learning target of reading comprehension. Moreover, the instruments used for the assessment of each aspect should fit the targeted context worldwide. Further instrument validation across contexts (i.e., EFL and ESL) are needed. The other limitation of this study lies in the age group, which was only limited to university students. Thus, additional studies that focus on younger students in primary and secondary schools, especially in the Indonesian context where English is a FL, should be conducted. In addition, although reporting is important for studies on learning motivation, the current study overlooked the metacognitive awareness of the students.

## 7 Pedagogical implications

Theoretically, it was expected that students of multilingual group would outperform those of the bilingual group in every construct of the motivational structure in our hypothesized model. As indicated in the result part, this study has several pedagogical implications which are important for teachers to pay attention to in the light of the two subsamples of bilingual and multilingual groups. First, reading motivation in both groups, particularly intrinsic motivation, can facilitate students' self-related belief (self-efficacy and self-concept with their ERC. Any attempt to improve students' reading motivation, especially intrinsic reading motivation can positively affect both the bilingual and multilingual students’ ERC. As indicated by group differences in the motivational variables, students with more FL mastery have the benefit of better self-concept and self-efficacy and are intrinsically more motivated to read in English. However, bilingual students tend to display more reading behaviors than those of multilingual students. Reading behavior is viewed as an indicator of reading motivation and facilitates intrinsic reading motivation with ERC. Therefore, inculcating the reading habit to intrinsically motivated students can help them optimize their reading comprehension achievement. This study also provides evidence that, although slightly significant, categorizing students based on previous FL learning experience can help teachers determine possible differences in the use of every variable employed in the motivational structure model. In this manner, teachers will know what to prioritize in their teaching to improve the reading comprehension achievement of students.

## Declarations

Ethics Approval Number: April 2022.

Name of the ethics committee: The Institutional Review Board (IRB) of the Doctoral School of Education, University of Szeged.

## Data availability statement

At this point, the data cannot be shared because it is a component of an ongoing study.

**Funding Statement**

This work was supported by the University of Szeged Open Access Fund (grant number: 6124) and the Research Programme for Public Education Development, Hungarian Academy of Sciences (grant KOZOKT2021-16).

## CRediT authorship contribution statement

**Helta Anggia:** Writing – original draft, Methodology, Investigation, Formal analysis, Data curation, Conceptualization. **Andrea Magyar:** Writing – review & editing, Validation, Supervision, Methodology. **Anita Habók:** Writing – review & editing, Visualization, Validation, Supervision, Methodology.

## Declaration of competing interest

The authors declare that they have no known competing financial interests or personal relationships that could have appeared to influence the work reported in this paper.

## Figures and Tables

**Appendix 1:** Testing invariance of the structural model across bilingual and multilingual groups

Paths (constraints)	*χ2*	*df*	Δ*χ2*	^a^P-value	CFI	^b^*Z-score*
Null model (no constraints)	4.740	4		0.315	1.000	
Structural weights	36.012	15		0.002	0.989	
Self-efficacy – Extrinsic Motivation			15.396	0.000		−3.937
Self-efficacy – Intrinsic Motivation			0.286	0.593		−0.535
Self-concept – Extrinsic Motivation			1.098	0.295		1.048
Self-concept – Intrinsic Motivation			0.236	0.627		0.486
Extrinsic motivation – ERB			2.201	0.138		−1.484
Intrinsic Motivation – ERB			9.223	0.002		3.043
ERB – English reading comprehension			1.163	0.281		−1.079
Extrinsic motivation – English reading comprehension			1.869	0.172		−1.368
Intrinsic Motivation – English reading comprehension			0.764	0.382		0.874
Self-efficacy – English reading comprehension			0.264	0.607		0.514
Self-concept – English reading comprehension			0.005	0.945		0.546
